# The Black Male Dementia Caregiver Study: An Analysis of the Relationship between Informal Caregiving, Sleep, Depression, and Cognitive Functioning

**DOI:** 10.1002/alz.092591

**Published:** 2025-01-09

**Authors:** Amani L Ali, Alexander Delong, Robert W Turner

**Affiliations:** ^1^ George Washington University School of Medicine and Health Sciences, Washington, DC USA; ^2^ George Washington University School of Medicine and Health Sciences, Washington, D.C., DC USA

## Abstract

**Background:**

Stress associated with caregiving for a person with Alzheimer’s Disease and Alzheimer’s Disease Related Dementias (AD/RD) has negative health implications. However, little is known about the implications of stress on non‐Hispanic Black (NHB) informal male caregivers. This study aims to examine the relationship between sleep, depression, and cognitive function in a sample of NHB informal male caregivers in the metropolitan Washington, D.C. area.

**Method:**

This cross‐sectional study (*n* = 68) included informal caregivers and non‐caregivers who participated in self‐perceived health questionnaires and a series of cognitive assessments. Data analysis consisted of logistic regression models to understand the relationship between caregiving, cognitive health, age, education, sleep (Pittsburgh Sleep Quality Index), and depression (Center for Epidemiological Studies Depression Scale).

**Result:**

Three variables were used to explore caregiving: caregiver status, caregiver tenure, and hours per week spent caregiving. Tenure was positively associated with hours per week spent caregiving (*p* = 0.031). Poorer sleep was associated with caregiver status (*p* = 0.030) and weekly time spent caregiving (*p* = 0.002), while depression was associated with caregiver status (*p* = 0.024). Additionally, there was an association between caregiver status and the Verbal Fluency Test (VFT) (*p* = 0.050) and the Verbal Naming Test (VNT) (*p* = 0.035), which became stronger when adjusting for sleep and depression (*p* = 0.023). Additional hours spent per week providing care was associated with poor performance on the Number Span Test (NST) (*p* = 0.012), which is exacerbated when adjusting for sleep and depression (*p* = 0.009). Caregiver tenure was associated with lower scores on the VFT (*p* = 0.003), unimpacted by adjusting for sleep or depression.

**Conclusion:**

In this study of NHB male informal caregivers, poor sleep was associated with lexical retrieval deficits, with caregiver tenure exacerbating this. Hours spent giving care per week is also associated with decreased sleep and attentivity. The results from this pilot study indicate a need for interventions to improve sleep quality among caregivers, thus reducing cognitive burden. Given this group’s unique cultural perspectives on sleep, curating targeted cognitive behavioral sleep interventions should be examined.

